# Optimization of the KNN Supervised Classification Algorithm as a Support Tool for the Implantation of Deep Brain Stimulators in Patients with Parkinson’s Disease

**DOI:** 10.3390/e21040346

**Published:** 2019-03-29

**Authors:** Gabriel Martin Bellino, Luciano Schiaffino, Marisa Battisti, Juan Guerrero, Alfredo Rosado-Muñoz

**Affiliations:** 1 Faculty of Engineering, National University of Entre Ríos: route 11, Km 10, 3100 Oro Verde, Argentina; 2 Faculty of Life and Health Sciences, Autonomous University of Entre Ríos, Alem 217, 3100 Paraná, Argentina; 3 Faculty of Science and Technology, Autonomous University of Entre Ríos, route 11, Km 10.5, 3100 Oro Verde, Argentina; 4 GDDP, Department of Electronic Engineering, School of Engineering, Universitat de Valencia, Burjassot, 46100 Valencia, Spain

**Keywords:** deep brain stimulation-DBS, microelectrode registers-MER, K-nearest neighbour-KNN algorithm, feature selection, Parkinson’s disease

## Abstract

Deep Brain Stimulation (DBS) of the Subthalamic Nuclei (STN) is the most used surgical treatment to improve motor skills in patients with Parkinson’s Disease (PD) who do not adequately respond to pharmacological treatment, or have related side effects. During surgery for the implantation of a DBS system, signals are obtained through microelectrodes recordings (MER) at different depths of the brain. These signals are analyzed by neurophysiologists to detect the entry and exit of the STN region, as well as the optimal depth for electrode implantation. In the present work, a classification model is developed and supervised by the K-nearest neighbour algorithm (KNN), which is automatically trained from the 18 temporal features of MER registers of 14 patients with PD in order to provide a clinical support tool during DBS surgery. We investigate the effect of different standardizations of the generated database, the optimal definition of KNN configuration parameters, and the selection of features that maximize KNN performance. The results indicated that KNN trained with data that was standardized per cerebral hemisphere and per patient presented the best performance, achieving an accuracy of 94.35% (*p* < 0.001). By using feature selection algorithms, it was possible to achieve 93.5% in accuracy in selecting a subset of six features, improving computation time while processing in real time.

## 1. Introduction

Parkinson’s disease (PD) is a chronic neurodegenerative disorder that is produced by the progressive death of the neurons producing dopamine in the substantia nigra. A decrease in dopamine levels produces an effect in other basal nuclei of the brain, such as the striatum, the subthalamic nucleus, and the globus pallidus, which play an important role in the inhibition and control of the human movements [1,2]. PD is a major health problem that affects approximately 1–2% of people over 65 years of age worldwide [3]. The motor symptoms of PD are bradykinesia, tremor at rest, rigidity, and postural instability. Non-motors symptoms are sleep disorders, cognitive dysfunctions, among others [1,2].

The initial treatment of PD is pharmacological; however, in some cases, an adequate control of the symptoms is not achieved. In other cases, the medication causes undesired side effects, such as the appearance of dyskinesia or intolerance. Also, after five or six years of this treatment, its effectiveness begins to diminish and the initial symptoms reappear [1,2]. In these cases, deep brain stimulation (DBS) is an effective treatment [4]. DBS is a therapy that involves the release of an electrical current in a deep part of the brain to treat a neurological dysfunction. This electrical current is formed by pulses of controllable amplitude, frequency, and voltage, which are provided by an implantable pulse generator and transmitted through a stimulation electrode, which acts as a pacemaker of the brain and it is effective in improving the motor disorders of PD. There are two deep brain structures whose stimulation has shown to have consistent therapeutic effects on the motor symptoms of the disease and dyskinesias that are produced by levodopa. These target structures are the subthalamic nucleus (STN) and the globus pallidus in its internal segment (GPi). However, the most common stimulation zone is the STN of both cerebral hemispheres to reduce the chronic hyperactivity of the neurons involved [5,6]. Stimulation of the STN allows for reducing the doses of pharmacological treatment of the patients and it requires less energy than the stimulation of the GPi. The STN is a discoid structure of approximately 5.9 × 3.7 × 5 mm^3^ that is located between the diencephalon and mesencephalon surrounded by substantia nigra, red nucleus, and zona incerta [2].

A multidisciplinary team with experience in the field is required to perform the implantation of electrodes, which is ideally formed by neurologists, neurophysiologists, neurosurgeons, and trained surgery personnel. Stereotactic procedures are used in order to achieve correct implantation, being combined with magnetic resonance imaging (MRI) and computed tomography (CT) during the procedure if the technology is available, and microelectrode recordings (MER) [6,7]. Normally, the patient is not anaesthetized to undergo surgery, in some cases, only sedation is needed, since it is necessary to evaluate the clinical response to the stimulation and reduce the side effects. A macro-stimulation device is used that releases a current in the target structure reached by the electrodes to perform the stimulation during the surgical procedure.

**Figure 1:**
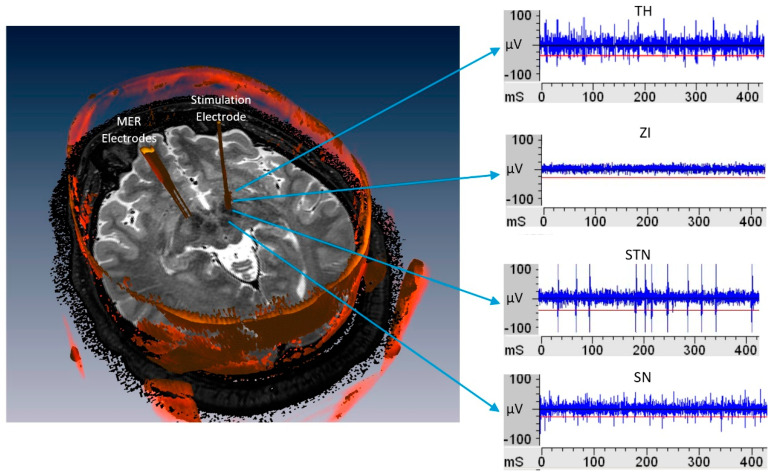
On the left, three-dimensional (3-D) view of the brain structure with microelectrode recordings (MER) trajectories to target marked. On the right, neural activity registered of different subcortical structures as the MER descends into the brain.

The analysis of the MER records is a widely used method of localization in deep structures, through visual and acoustic analysis and beta rhythm analysis [4]. MER electrodes has less than 100 micrometers diameter and it goes into the brain through different functional structures, such as the anterior thalamus (TH), zona incerta (ZI), subthalamic nucleus (STN), and substantia nigra pars reticulata (SNr) [4]. Each one of them presents a specific neural activity, as seen in Figure 1, constituting non-stationary signals [4,6], conformed by: the neuronal activity called spikes, the background neuronal activity, and artifacts [8].

The localization of the STN for implantation of DBS electrodes is complex task for neurophysiologists, as presented in [4,9], a combination of anatomic localization via stereotactic procedures with functional localization via MER is used to achieve a correct implantation as well as to identify the best implantation zone in the STN. It has been proven that MER readings can reduce targeting errors that are related to the resolution limitations of the MRI and CT images, and the anatomic shifts during surgery [9]. In this context, a brain shift is produced when the burr holes are made [6]. The MERs signals amplitudes can vary from 50 µV up to 200 µV [8,9,10], and they are visually and acoustically analyzed, which requires a clean and crisp signal. Accordingly, signal conditioning must be done before starting the records, while using the pre-amplification module and software gain and filters in the set-up stage. In this stage, environmental interference or noise issues are solved.

In the last 10 years, several works have been reported [4,11], where signal processing and data mining have been used to locate specific regions of the brain, such as TH, ZI, STN, or SNr, which are solely based on MER information.

Chaovalitwongse et al. [4] proposed three classifiers: Naive Bayes, k-nearest neighbors (KNN), and decision trees, which all use 13 temporal features estimates from MER. In this work, the authors obtained 82.2% accuracy for the KNN algorithm, 82.8% accuracy with the Bayesian kernel, and 89.6% accuracy with the classification trees. In addition, a time-frequency indicator was used to train KNN and decision trees, but the obtained results were not encouraging (50.2% average accuracy). The Bayesian approach takes into account the posterior conditional probability that was calculated from each and every feature. Thus, noisy features can play a role in reducing classifier accuracy. In the classification trees algorithm, the variables and thresholds that were used at each branching point are independent of each other. This ensures that the algorithm always chooses the variable that is best suited for partitioning the set. The algorithm prioritizes the importance of each feature based on the hierarchical structure of decision-making. The result of [4] confirm that identifying the most informative features in distinguishing different subcortical structures is necessary, but the authors do not explore feature selection algorithms to choose which ones are more suitable for their classification process.

Rajpurohit et al. [12] worked with four classification methods: Logistic Regression (LR), Gaussian Naive Bayes (GNB), KNN, and support vector machine (SVM). In this case, the author and colleagues extracted 13 temporal features from 65 MER tracks for classifier training and then used sequential forward and backward feature selection. Of the 13 temporal features, six were spike-detection-independent and seven were spike-detection-dependent. When using the entire set of normalized features with patient-independent normalization, the baseline classification error rate was approximately 17–19% for each of the four supervised learning classifiers. The resulting accuracy from each classification method was: LR 83%, GNB 84%, KNN 81%, and SVM 83%. The Logistic Regression algorithm does not show different weights to the characteristics according to their relevance and this can limit the performance of the classifier. The SVM classifier takes a long time to train but it achieves good results with the linear kernel. Occasionally, the SVM algorithm presents non-convergence problems.

In [4,12], the KNN algorithm was used, but only the Euclidean distance is explored. Likewise, no study is carried out to determine the optimal value of the number of neighbors in order to improve the performance of the classifier.

Cagnan et al. [11] proposed a classification method with a structure that was similar to a binary decision tree, working with two temporal features and two frequency variables that were linked to the power in the beta and gamma frequency range, with the aim of detecting the entry and exit of the electrode in STN. The algorithm achieved 88% accuracy in the detection of STN according to the classifications that were performed by neurophysiologists.

The works described above [4,11,12] used a debugged database without noisy records for the validation process of the classifiers. Consequently, the reported results are likely to yield higher performance values than the results that were obtained with those algorithms while using real MER records, as directly obtained in a DBS surgery.

In exploratory tests that were carried out with individual supervised classifiers, the KNN algorithm presented the best performance with our database. In this article, we present a KNN based algorithm that was trained and validated with different features that were calculated from MER records, in order to identify the STN as a subcortical structure target for DBS in PD. We explore how to optimize the performance of KNN in order to achieve clinical relevance for the application of the algorithm as a support tool in real time during DBS surgeries. For the clinical purpose sought, our hypothesis suggests that the optimization of the KNN classifier can be achieved with the combination of three aspects: the different standardizations of the calculated features, the optimal definition of KNN configuration parameters, and the application of algorithms for adequate feature selection.

## 2. Materials and Methods

### 2.1. Database

The records of neuronal electrical activity at different depths of the brain were obtained from bilateral DBS surgery that was performed in 14 non-medicated patients with PD, aging 57 ± 6 (eight male/six female), where the stimulator was implanted in the STN. These surgeries were performed at the Hospital La Fe in Valencia, Spain. All of the patients met medical accepted selection criteria and signed an informed consent for DBS surgery with MER. These investigations that use human data were carried out following the rules of the Declaration of Helsinki. The Ethical Committee for Biomedical Research of La Fe Hospital approved the research procedures for the project with registration number 2015/0824 in May 17, 2016.

For surgical planning, T1 and T2 weighted 1.5T MRI series were fused with a CT scan that was performed after placing the stereotactic frame. As per standard clinical protocol, the target coordinates and trajectory to the STN were identified while using the fused images on a neuro-navigational platform (StealthStation, Medtronic Corp, Minneapolis, MN, USA). Two neurophysiologists continuously analyzed the electrical records that were obtained with MERs as the electrode goes through different structures of the brain, followed by standard stereotactic techniques. This procedure allowed for determining whether the brain structure is STN, defining the entry and exit limits, as well as identifying the best implantation zone within this basal structure. This classification was validated off-line by fused images of the intraoperative CT with the preoperative images. In our work, the record of neuronal electrical activity was performed for each cerebral hemisphere, with two platinum-iridium (Pt-Ir) MER per hemisphere that entered parallel and were separated by 2 mm each other. Medtronic MER with an impedance of 1 MΩ at 1000 Hz was used. The records were obtained at a sampling frequency of 12 kHz with a 16-bit converter, a total gain of 10,000, applying a line filter at 50 Hz, and a Butterworth band pass filter between 200 Hz and 6000 Hz. All of the records were obtained with the Alpha Omega MicroGuide Pro^TM^ system. This system also acquired the depth of the MER in [mm] respect to the surface of the skull and the position that was marked as target. Records started 7 mm before the target area with an advance of the MER electrodes in steps of 0.2 mm, recording at least 16 s in each position.

### 2.2. Features Obtained from MER Records

Signals were processed in 4 s windows that overlapped 50% in order to calculate 18 temporal features, in an off-line analysis using Matlab^®^ version R2017a. It was demonstrated in [4] that this window size is optimal in capturing enough spikes to detect changes in the subcortical structures and small enough for real-time processing. The records were used as they were exported from the Alpha Omega MicroGuide Pro^TM^ equipment (Nazareth, Israel). Only the records that were acquired during the time when the MER was descending from one position to another were eliminated, since they were heavily noisy. For valid records, the following features were calculated as proposed in the state of art [4,13]:

Spike-detection-independent features:

- Basal amplitude value (1-VAB): Dolan et al. [14] proposed a robust method using the Hilbert transform to estimate the envelope of a time record MER using Equation (1), where E(t) is the envelope and H{X(t)} is the Hilbert transform of the signal X(t).
  (1) $$E\left(t\right)=\sqrt{X{\left(t\right)}^{2}+(HT{\left\{X\left(t\right)\right\}}^{2}}\;,$$
- Root mean square amplitude (2-RMS): Calculated according to Equation (2) [4].
  (2) $$RA=\sqrt{\frac{\sum_{i=1}^{n}{\left(X_{i}\right)}^{2}}{n}}\;,$$
- Kurtosis (3-KUR): Statistically, it is a measure that is used to describe a distribution. Whereas, skewness differentiates the extreme values in one versus the other tail, kurtosis measures extreme values in either tail. If the distributions of the variables are not known and discrete data is available, *K* can be estimated according to Equation (3), where $\mu_{4}$ is the fourth moment with respect to the mean and σ the standard deviation.
  (3) $$K=\frac{\mu_{4}}{\sigma^{2}}\;,$$
- Curve length (4-CL): Calculated according to Equation (4) [4]. Where $x_{i}$ is ith point data vector of length n.
  (4) $$CL=\sum_{i=1}^{n-1}\left|X_{i+1}-X_{i}\right|\;,$$
- Threshold (5-TH): Calculated according to Equation (5) [4]. Where μ is the mean of the data vector.
  (5) $$TH=\frac{3}{n-1}\sqrt{\sum_{i=1}^{n}{\left(X_{i}-\mu \right)}^{2}}\;,$$
- Peak count (6-PK): Calculated according to Equation (6) [4].
  (6) $$PK=\frac{1}{2}\sum_{i=1}^{n-2}\max\left\{0,sgn\left[X_{i+2}-X_{i+1}\right]-sgn\left[X_{i+1}-X_{i}\right]\right\}\;,$$
- Average nonlinear energy (7-NE): Calculated according to Equation (7) [4]. High values of NE show the presence of high frequency signals in the segment’s analysis.
  (7) $$NE=\frac{1}{n-2}\sum_{i=2}^{n-1}[{X_{i}}^{2}-X_{i-1}X_{i+1}\;]\;,$$
- Zero crossings (8-ZC): Calculated according to Equation (8) [4].
  (8) $$ZC=\frac{1}{2}\sum_{i=1}^{n-1}sgn\;(X_{i+1})-sgn(X_{i})\;\;,$$

Spike-detection-dependent features:

- Spike burst index (9-SBI): ratio of the number inter-spike intervals (ISIs) less than 10 ms to the number that is greater than 10 ms [15].
- Spike pause index (10-SPI): ratio of the number of ISIs greater than 50 ms to the number less than 50 ms [15].
- Spike pause ratio (11-SPR): ratio of cumulative time of ISIss greater than 50 ms to the cumulative time of those less than 50 ms [15].
- Spike count (12-SC): instead of using the spike count, in this work, we proposed to use spike frequency, in spike per seconds of the segments analyzed.
- Mean spike amplitude differential (13-SMAD): 80 percent trimmed mean of the difference between consecutive spike amplitudes [4].
- Spike count ratio (14-SCR): fraction percentage of spikes accepted as genuine spikes among candidate spikes by the spike detector [4].
- Median of the spike count (15-SF): Calculated as the median of SC.
- Standard deviation of the ISIs (16-SSD) [4].
- Mean value of the ISIs (17-SDp).
- Median value of the ISIs (18-SDm).

The database was formed with 34,898 records of 4 s windows, in which we calculated the 18 features listed above, and each window was labeled as STN or non-STN according to the data that was provided by trained neurophysiologists and co-register images. In this context, 52% of the total windows corresponded to the STN class. From this database, three new databases were generated with the same number of records as the original in which different standardizations were applied to each feature. Subsequently, a standardization of the original database was made, subtracting the mean and dividing by the standard deviation of the features that were selected by these criteria:

- Each feature was standardized based on the values of that feature calculated for all patients.
- Each feature for patient was standardized based on the values of that feature calculated for own patient.
- Each feature for patient and hemisphere was standardized based on the values of that feature calculated for own patient and each cerebral hemisphere.

### 2.3. K-Nearest Neighbors Classifier (KNN)

Let *R*(*z*) ⊂ ℜ^*N*^ be a hypersphere with volume *V* and center *z*. *N_k_* is the number of samples of the training set *T_k_* for the classifier KNN and *W_k_* the class assigned. The probability of having exactly *n* samples within *R*(*z*) has a binomial distribution [16], according to (9).

(9) $$E\left[n\right]=N_{k}\int_{y\;\in R\left(z\right)}p(y\;|\;w_{k})\;dy\;\approx N_{k}V_{p(y|w_{k})}\;\;,$$

If a radius is set around *z* that generates a volume that contains exactly *K* samples, then that radius and its volume depend on the position *z* in the measurement space [16]. Therefore, we can write *V*(*z*) instead of *V* being the estimate of density [17], as indicated in (10).

(10) $$\hat{p}(z\;|\;w_{k})=\frac{K}{N_{k}V\left(z\right)}\;\;,$$

In the KNN algorithm, the *K* value is set to estimate the model and the minimum volume *V*(*z*) that these *K* samples cover is calculated. The expression (2) indicates that, in the regions where the density estimate is large, the volume is expected to be small. If the estimate is small, then the sphere needs to grow to collect the necessary samples [17]. Moreover, the *K* parameter controls the balance between bias and variance, as indicated in (11) [16].

(11) $$\begin{array}{c} K\to \;\infty \;y\;N_{k}\to \;\infty \;,\;\mathrm{in}\;\mathrm{order}\;\mathrm{to}\;\mathrm{get}\;a\;\mathrm{small}\;\mathrm{variance};\\ \frac{K}{N_{k}}\;\to \;0\;y\;N_{k}\to \;\infty \;,\;\mathrm{in}\;\mathrm{order}\;\mathrm{to}\;\mathrm{get}\;a\;\mathrm{small}\;\mathrm{bias} \end{array}$$

The KNN technique has a practical interest, since it works on the set of samples to estimate the model without calculating the probability density. If *K_k_* is the number of neighboring samples that are found in the *W_k_* class, then a conditional density estimator is (12).

(12) $$\hat{p}(z\;|\;w_{k})\approx \frac{K}{N_{k}V\left(z\right)}$$

By combining (2.12) with the Bayes Naive classifier with a uniform cost function [16], it is possible to obtain the estimated classification according to (13).

(13) $$\begin{array}{c} \hat{w}\left(z\right)=w_{k};\\ k=\arg\underset{i=1,\ldots ,k}{\max}\left\{\hat{p}\left(z|w_{i}\right)\hat{P}\left(w_{i}\right)\right\};\\ k=\arg\underset{i=1,\ldots ,k}{\max}\left\{\frac{K_{i}}{N_{i}V\left(z\right)}\frac{N_{i}}{N_{s}}\;\right\};\\ k=\arg\underset{i=1,\ldots ,k}{\max}\{K_{i}\} \end{array}$$

From the previous expression, it is concluded that the class that is assigned to the vector *z* is that with the largest number of neighbour samples of the class *W_k_* closest to *z*. In this work, Bayesian optimization is applied to minimize the classification error with our database, and thus to determine the optimal configuration of KNN. In this work, a value of *K* = 9 and a city block distance metric were adopted for KNN algorithm.

### 2.4. Performance of KNN Classifiers

For the training and validation process, 14 different subsets were generated for each of four databases (original and three with different standardization, as described in Section 2.2) in order to simulate a real situation as during a DBS surgery. In each subset, subset 1, for example, the data from patient 1 was taken for validation and data from the rest of the patients (2 to 14) for training and then, for the other subsets, the same procedure was applied, as proposed in [4]. This method is known as leaving one patient out. In this way, 14 KNN classifiers were calculated with each database with non-standardized (KNN) features, and standardized features: with data from all patients (KNN_STA); with data per patient (KNN_PAT); and, with data per patient and per cerebral hemisphere (KNN_HEM). Subsequently, using each respective validation datasets, the performance of the classifiers were analyzed by calculating the performance indices that were widely reported in the state of art [4,12,18]: accuracy (ACC), sensitivity (SEN), specificity (ESP), area under the ROC curve (AUC), and index of diagnosis (DOR).

A descriptive statistic study was carried out and statistical comparisons were made with nonparametric tests, after checking for assumptions of normality (Kolmogorov–Smirnov test; p<0.05). Friedman test was used for the analysis of global significance, and for the paired comparisons of classifiers, Nemenyi was used as a post-hoc test. The threshold of significance between the comparisons was accepted at 95% (p<0.05). All of the results are expressed as mean and standard deviation (Mean ± SD). SPSS Statistics v24 and Statistics and Machine Learning toolbox for Matlab^®^ version R2017a were used to calculate the performance indexes and statistical analysis.

### 2.5. Feature Selection Techniques

Feature selection for classification can be defined as a combinatorial optimization problem, where a set of features is selected in a way that maximizes the quality of the hypothesis that was learned from these features. Supervised methods of feature selection can be categorized in filter models and wrapper models [19], where the methods are not intrinsic to the classification algorithm.

Filter models separate the feature selection from the classifier learning, so that the bias of a learning algorithm does not interact with the bias of a feature selection algorithm [20]. It is based on measurements of the general characteristics of training data, such as distance, consistency, dependence, information, and correlation. These methods can be univariate if they do not take the values of other attributes when the selection procedure took place into account, or multivariate if they consider the interaction of the other characteristics [20]. Robnik-Sikonj et al. [21] related ReliefF’ s relevance evaluation criterion to the margin maximization hypothesis, concluding that ReliefF provides superior performance in many applications, being a more stable algorithm than other filter type algorithms [22]. ReliefF (RS), being multivariate, also allows for detecting redundant features, which is why it was selected in the present work as a filter type selection method [22].

Kononenko et al. in 1994 proposed relief and RS class extension [21]. Basically, the method consists of selecting features randomly and then, depending on the closest neighbors, assigning more weight to the features that better discriminate among classes. When considering that instances are randomly sampled from data, then the score of the *i*-th feature *S_i_* is defined according to Equation (14), where *M_k_* denotes the values in the *i*-th feature of the closest instances *X_k_* with same class tag, while *H_k_* denotes the values in the *i*-th function of the instances that are closest to *X_k_* with different class tags, and *d*(·) is a measure of distance [21].

(14) $$S_{i}=\frac{1}{2}\sum_{k=1}^{l}\left[d\left(X_{ik}-X_{iM_{k}}\right)-d\left(X_{ik}-X_{iH_{k}}\right)\right]$$

In the wrapper model, the searching procedure for subsets of features consists in generating several subsets that were obtained from the original set that are evaluated while using a classifier algorithm [22]. It is important to note that the number of subsets increases exponentially as the number of features increases, and it is necessary to use heuristic methods to guide the search, making these methods somewhat challenging [22]. Generally, wrapping methods have better performance than filter methods [19], since improving the classification algorithm also improves the process of feature selection. However, wrapping methods can be computationally more expensive for problems with large dimensions, greater than 50 features, since each subset of features considered must be evaluated by the classification algorithm [22]. In this work, we used three wrapper model algorithms that were applied to the KNN classifiers described in the previous sections: backward, forward, and branch and bound. Once the classifier is selected, a wrapping model will perform the following steps:

- Step 1: Look for a subset of features,
- Step 2: Evaluate the subset of features selected by the performance of the KNN classifier, and
- Step 3: Repeat Step 1 and Step 2 until reaching the desired performance.

The feature search methodology produces a subset of features that are used to train the classifier. The resulting classifier is then evaluated with an independent data set that has not been used in the training process [22]. As mentioned before, several heuristic search strategies were used, such as: backward elimination, forward selection, and branch and bound [19]. In the forward technique, search begins with an empty set of features and then progressively incorporates features into a larger subset. In the backward technique, it begins with a set that conformed to all features and those that do not contribute to the performance of the classifier are progressively eliminated. In branch and bound method, the algorithm starts from the full set and it removes the features through a first deep search with a reverse strategy. In this last strategy, a search is systematically carried out by means of a tree structure when considering that nodes whose objective function are lower than the current best ones are not explored, under the assumption of monotony that ensures that their sons will not contain a better solution. The branch and bound method (BBS) has shown that it does not fall into local minimums, such as backward (BS) and forward (FS), given the algorithm’s search nature [19]. The feature selection algorithms that were described in this section were applied to one database selected based on best performance indicators.

### 2.6. Performance of KNN Classifiers with Feature Selection

The same sets of training and testing data that are detailed in Section 2.4 were used for KNN classification with all features (KNN) and with the features resulting after applying feature selection methods, such as ReliefF (KNN+RS), backward (KNN+BS), forward (KNN+FS), and branch and bound (KNN+BBS). Thus, the results that were obtained of each classifier are comparable.

We analyzed KNN+RS, KNN+BS, KNN+FS KNN+BBS, and KNN, as five different classifiers. For each classifier, the performance indices detailed in Section 2.4 were calculated: SEN, ESP, ACC, and AUC. We carried out the non-parametric Friedman test, followed by Nemenyi post-hoc test for pairwise comparisons if the results of the Friedman test indicated overall significance (p<0.05), as presented in Section 2.4.

## 3. Results and Discussion

### 3.1. Results of the KNN Classifiers

Table 1 shows the average results that were obtained from the 14 classifiers for the four versions of the KNN algorithm according to Section 2.4. Likewise, Table 2 presents the average training and validation times. The results indicate that all four classifiers train quickly and at similar times. On the other hand, the validation process for the data of a new patient represents an order of magnitude higher than the training time.

**Figure 2:**
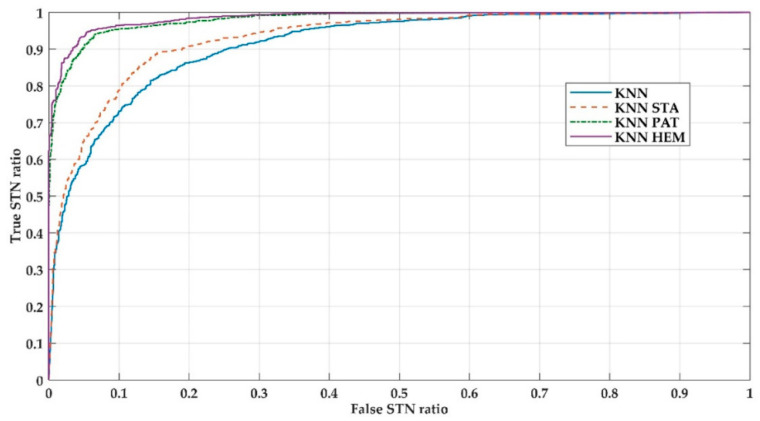
ROC Curves for the 4 versions of the KNN algorithm.

Figure 2 presents the ROC curves for the four versions of the algorithm. It can be seen that both the average values of Table 1 and the area under the ROC curve of Figure 2 show that the KNN_HEM version presents the best average performance for all of the indicators.

The obtained results for the four trained classifiers were statistically compared by Friedman and each performance indicator. Given that, in all cases, the test showed global significance (p<0.001) and the Nemenyi test was performed to obtain pairwise comparisons, whose results are presented in Table 3.

The standardization of features by patient and by hemisphere (KNN-HEM) allowed for raising the mean accuracy by 15% when compared with the KNN that was trained with the database without standardized (KNN) and 10% higher when compared with KNN trained with the database standardized of all patients (KNN-STA). From the analysis of the previous results, it is observed that, despite all the trained KNN classifiers showing an acceptable performance, KNN_HEM presents a significant improvement when compared with KNN (p<0.001) and KNN_EST (p<0.001). This fact shows that, even under the experimental conditions as the present work, standardizing the data per patient and per cerebral hemisphere allows for us to obtain encouraging results. Analyzing the increase in performance indices according to the standardization method that is used reflects this improvement.

Our study obtained 94.35% mean accuracy while using the KNN-HEM algorithm, while Rajpurohit et al. [12] obtained 81% accuracy and Chaovalitwongse et al. [4] obtained 82.2% accuracy using KNN. In addition, the best accuracy values that were obtained in Rajpurohit et al. [12] were obtained with the GNB classifier, obtaining an average accuracy of 84%. In the work by Rajpurohit et al. [12], in which 10 cross-validation was used as a data partition for training and testing, bias is very likely to occur in favor of the performance accuracy of the classifiers being used. Chaovalitwongse et al. [4] obtained the best performance with a classifier tree, obtaining an average accuracy of 89.6%

Although it is not possible to make a direct comparison with the results that were obtained in other works, since different databases are used, the classification percentages obtained in the present work with KNN_HEM are higher than those that were obtained by [4,11]. It is observed from Table 1 that, in all cases, the sensitivity is superior to the specificity, which indicates that the KNN algorithm has greater capacity to detect the STN area, which presents clinical relevance in the context of the application.

### 3.2. Results of the KNN Classifiers with Feature Selection

Table 4 and Table 5 present the selected features for each feature selection algorithms, showing the results of the performance indicators for each classifier trained with selected features for each algorithm along with the KNN trained with all features. As presented, KNN+RS and KNN+BBS trained with seven and six features respectively, of which five of them are the same, accuracy, as other indicators, are slightly lower than KNN (0.98% for KNN+RS and 0.9% for KNN+BBS). It is noteworthy that the number of features is one-third of the 18 features, which facilitates the process of classification in real time. KNN+BS (trained with 12 features) and KNN+FS (trained with 11 features) slightly improve the performance indicators when compared with KNN, 1.21% and 1.13%, respectively, higher for accuracy.

Table 6 presents the results of Friedman and Nemenyi tests for each classifier with feature selection and for each performance indicator. It can be observed that, statistically, KNN+BS and KNN+RS models present significant differences for all of the indicators with KNN+RS and KNN+BBS. KNN+FS does not present statistically significant differences for any performance indicator when compared with KNN. In the case of KNN+BS, there are two indicators that show statistically significant differences with KNN: ACC (p=0.047) and SEN (p=0.013).

The performance indicators for the different models that were obtained with feature selection do not present average percentage differences that indicate a clinical relevance. From this perspective, it is more important to achieve models that require a smaller number of features to be calculated during a surgery, and thus guarantee a classification in a shorter time. In the case of the models with fewer features, (KNN + RS and KNN + BBS), KNN + BBS has a higher value than KNN + RS for three of the four performance indicators, and at the same time, KNN + BBS does not present statistically significant differences with KNN for all of the indicators. The branch and bound method managed to select this subset of features that is present in all other feature selection algorithms, resulting in an adequate combination of four features that are linked to the background activity (4-CL, 5-TH, 6-PK, and 8-ZC) and two linked to the spikes (13-SMAD and 14-SCR). These results are concordant with that obtained by Rajpurohit et al. [12], which, when applying the features selection by wrapping methods (backward and forward), managed to improve the performance of the KNN classifiers, logistic regression, SVM, and Bayesian developed in their work. In the case of KNN, which has the lower error in this work, it was trained with seven features out of a total of 13, five of them being related to the background activity spike-detection-independent and two features that are related to the activity of spikes.

Other authors, such as Novak et al. [9], reported that the background activity in the STN of 15 patients presented a statistically significant difference between the STN, TH, and ZI, as well as with substantia nigra. In this paper, they also reported that the TH amplitude of background activity is similar to the substantia nigra. Przybyszewski et al. [23] reported that, in a study with 10 patients with statistical validation, the background activity of multiple unit activity in combination with the average power in the beta range of local field potentials had discriminating power to detect the STN of nearby structures. In both studies, it was concluded that having features linked to the background activity is important in the detection of STN. 

In regards to feature selection techniques, all of the selection methods used conferred more importance to the features that are associated with the background activity. In particular, 4-CL, 5-TH, 6-PK, and 8-ZC are present in all of the results obtained by the different feature selection methods, possibly those that best represent the background activity. At the same time, features 13-SMAD and 14-SCR, which are associated with the spikes, were also selected by all methods, showing that they better characterize the activity of the spikes.

## 4. Conclusions

The results of the present work initially propose a KNN_HEM model that constitutes a first step for an automatic classification system that works in the operating room as a support tool for neurophysiologists and neurosurgeons when defining the optimal location in the fixation of the stimulation electrode of a DBS system in patients with PD. The computational times that were obtained in training and validation imply that a KNN algorithm can be used to validate the data of a new patient in real time. A system of these characteristics will allow for the reduction of times of a surgery of this nature, providing an objective classification result.

Initially, 18 temporal features reported in the state of the art were adopted [4,11,12], with good results in the supervised classification. Later, investigating the algorithms of feature selection to those that could optimize the classification process with the data available in the database that was used is a future direction. The feature selection methods that were addressed in the present work allowed for obtaining classifiers with a performance that is similar or superior to the classifiers trained with all of the characteristics, eliminating those redundant or noisy features. It is noteworthy that obtaining trained classifiers with a smaller number of characteristics not only improves the classification time of the validation data, but also the calculation time of the characteristics from the MER records, thus improving the full computation time of the whole process.

Background activity represents small action potentials, with noise order levels, which are generated by neural populations near the recording electrode but not in direct contact with it, this describes the neuronal behavior of an STN volume. This could characterize neuronal population and allows, through supervised classification, the detection of registers coming from that basal nucleus. Neurophysiologists also identify this zone based on the spikes, analyzing both their amplitude and firing frequencies, which correspond to the action potentials of a group of neurons that is in contact with the electrode.

There is a lower capacity of the KNN algorithm as well as those that were reported by other authors [11,12] to differentiate the STN based on this information. Based on the results obtained and under the experimental conditions of the present work, it is proposed to use KNN + BBS in the classification process in real time during DBS surgeries.

## Figures and Tables

**Table 1:** Average values for accuracy (ACC), specificity (ESP), sensitivity (SEN), area under the ROC curve (AUC), and index of diagnosis (DOR) performance indices of the four versions of proposed k-nearest neighbors (KNN) classifiers.

KNN Version	ACC	ESP	SEN	AUC	DOR
KNN	0.8194 ± 0.0074	0.7863 ± 0.0114	0.8499 ± 0.0084	0.9028 ± 0.0048	21.9095 ± 2.1969
KNN_STA	0.8563 ± 0.0058	0.8299 ± 0.0090	0.8807 ± 0.0067	0.9230 ± 0.0033	36.1316 ± 3.5980
KNN_PAT	0.9358 ± 0.0033	0.9344 ± 0.0041	0.9371 ± 0.0064	0.9761 ± 0.0021	213.8659 ± 24.6348
KNN_HEM	0.9435 ± 0.0022	0.9422 ± 0.0042	0.9446 ± 0.0049	0.9815 ± 0.0019	279.8760 ± 22.9661

**Table 2:** Average values for training and validation in seconds of the four versions of the proposed KNN classifiers.

KNN Version	t_Train	t_Validation
KNN	0.0514 ± 0.0579	0.4044 ± 0.0221
KNN_STA	0.0369 ± 0.0022	0.4093 ± 0.0306
KNN_PAT	0.0349 ± 0.0020	0.4037 ± 0.0296
KNN_HEM	0.0357 ± 0.0038	0.4028 ± 0.0283

**Table 3:** *p*-values of the Friedman and Nememyi test. In bold, statistically significant differences.

KNN versions Compared	ACC	ESP	SEN	AUC	DOR
Friedman Test	**<0,001**	**<0.001**	**<0.001**	**<0.001**	**<0.001**
KNN vs. KNN_STA	0.1701	0.1701	0.1701	0.1701	0.1701
KNN vs. KNN_PAT	**<0.001**	**<0.001**	**<0.001**	**<0.001**	**<0.001**
KNN vs. KNN_HEM	**<0.001**	**<0.001**	**<0.001**	**<0.001**	**<0.001**
KNN_STA vs. KNN_PAT	0.1701	0.1701	0.1243	0.1701	0.1701
KNN_STA vs. KNN_HEM	**<0.001**	**<0.001**	**<0.001**	**<0.001**	**<0.001**
KNN_PAT vs. KNN_HEM	0.1701	0.1701	0.2945	0.1701	0.1701

**Table 4:** Selection for each algorithm.

RS	BS	FS	BBS
4-CL	1-VAB	2-RMS	4-CL
5-TH	2-RMS	3-kur	5-TH
6-PK	3-kur	4-CL	6-PK
8-ZC	4-CL	5-TH	8-ZC
12-SC	5-TH	6-PK	13-SMAD
13-SMAD	6-PK	7-NE	14-SCR
14-SCR	7-NE	8-ZC	
	8-ZC	9-SBI	
	9-SBI	12-SC	
	12-SC	13-SMAD	
	13-SMAD	14-SCR	
	14-SCR		

**Table 5:** Measure for each model with feature selection. In bold, the highest values that were obtained for each indicator.

Performance Measures	KNN + RS	KNN + BS	KNN + FS	KNN + BBS	KNN
Accuracy (%)	93.43 ± 0.36	**95.49 ± 0.27**	95.42 ± 0.36	93.50 ± 0.34	94.35 ± 0.22
Specificity (%)	93.45 ± 0.55	95.21 ± 0.35	**95.22 ± 0.38**	93.27 ± 0.51	94.23 ± 0.42
Sensitivity (%)	93.40 ± 0.65	**95.74 ± 0.49**	95.60 ± 0.56	93.72 ± 0.55	94.46 ± 0.49
AUC (%)	97.50 ± 0.23	**98.68 ± 0.14**	98.67 ± 0.17	97.58 ± 0.19	98.15 ± 0.19

**Table 6:** *p*-values of Friedman and Nemenyi tests. Nemenyi test compares the classifiers by pairs including the models with all features as those resulting from the selection of features, as described in Section 2.6. In bold, statistically significant differences.

KNN Versions Compared	ACC	ESP	SEN	AUC
Friedman Test	**<0.001**	**<0.001**	**<0.001**	**<0.001**
KNN+RS vs. KNN+BS	**<0.001**	**<0.001**	**<0.001**	**<0.001**
KNN+RS vs. KNN+FS	**<0.001**	**<0.001**	**<0.001**	**<0.001**
KNN+RS vs. KNN+BBS	0.996	0.974	0.952	0.875
KNN+RS vs. KNN	0.055	0.164	0.133	**0.024**
KNN+BS vs. KNN+FS	0.989	1.000	0.975	0.999
KNN+BS vs. KNN+BBS	**<0.001**	**<0.001**	**<0.001**	**<0.001**
KNN+BS vs. KNN	**0.047**	0.069	**0.013**	0.118
KNN+FS vs. KNN+BBS	**<0.001**	**<0.001**	**<0.001**	**<0.001**
KNN+FS vs. KNN	0.153	0.094	0.074	0.065
KNN+BBS vs. KNN	0.134	0.035	0.485	0.251
